# Validation of Biosensors for Monitoring Feeding Behaviors in Hanwoo Cattle

**DOI:** 10.3390/bios16100558

**Published:** 2026-10-03

**Authors:** Andrian Ragandang, Hyunjin Cho, Miae Ko, Seongwon Seo

**Affiliations:** 1 Department of Animal Biosystem Sciences, Chungnam National University, Daejeon 34134, Republic of Korea; ragandangian@gmail.com (A.R.); chohyunjin0927@gmail.com (H.C.); 2 Institute of Agricultural Science, Chungnam National University, Daejeon 34134, Republic of Korea; magogene67@gmail.com

**Keywords:** eating time, ruminating time, Nedap SmartTag neck, RumiWatch noseband sensor, Hanwoo, biosensor

## Abstract

Detecting feeding behavior in cattle is important but can be cumbersome when done manually. This study aimed to validate two wearable biosensors─RumiWatch noseband (RW) and Nedap SmartTag Neck (ND)—for monitoring feeding behavior in Hanwoo cattle. Specifically, we assessed the agreement between feeding behaviors recorded by RW and ND and those obtained manually through video observation (VO), focusing on eating time (ET) and ruminating time (RT). Three Hanwoo cows (540 ± 24.12 kg) were fitted with both sensors and continuously video-recorded for three consecutive days. Data from both 1 h (*n* = 216) and 24 h (*n* = 9) summaries were used to compare RW with VO, while ND data were analyzed only at the 24 h level. The RW showed strong agreement with VO for RT, with concordance correlation coefficients (CCC) of 0.98 (1 h) and 0.95 (24 h). In contrast, RT from ND showed poor agreement with VO (CCC = 0.12 for 24 h). Least squares means for RT (min/24 h) did not differ between VO (460 min) and RW (461 min), but ND significantly underestimated RT (211 min; *p* < 0.001). Both RW and ND performed poorly in detecting ET (CCC = 0.05 for RW and −0.15 for ND at 24 h). However, when time spent on both eating and other jaw movements was combined in VO, the combined time did not differ significantly from ET reported by RW (441 min vs. 454 min; *p >* 0.05), with improved agreement (CCC = 0.62). Cow, day, and their interaction had no significant effect on the deviation between VO and RW for RT (*p >* 0.05). Overall, these results indicate that RW accurately detects RT but tends to overestimate ET, whereas ND significantly underestimates both ET and RT with poor concordance in Hanwoo cows.

## 1. Introduction

Feeding behaviors, including eating and ruminating, are key determinants of cattle performance. In particular, ruminating time, which has a complementary relationship with eating time [1], is considered a potential predictor of milk yield and solids [2], with longer ruminating time being associated with higher milk yield [3]. Ruminating time is also closely linked to dry matter intake [4] and is used as an indicator of the health status of cattle [5,6].

To record these behaviors, continuous visual observation, either in real time or using a recorded video, has traditionally been used [7]. However, visual observation (VO) requires substantial labor and time [8,9] and is prone to human error [10]. With the advent of Precision Livestock Farming (PLF), which incorporates advanced farming technologies [11,12], these behaviors can now be recorded automatically with minimal labor. Wearable biosensors such as RumiWatch noseband (Itin + Hoch GmbH, Liestal, Switzerland; RW) and Nedap SmartTag Neck (Nedap Livestock Management, Groenlo, The Netherlands; ND) are among these technologies. The RW enables automated measurements of ruminating, eating and drinking behaviors based on jaw movements [13]. The ND uses a triaxial accelerometer (G-sensor) to detect eating time based on head movements and the periodic jaw movements associated with regurgitation and subsequent chewing of the bolus during rumination, in both lying and standing positions [14,15].

Various validation studies have been conducted to assess the ability of the RW and ND systems to detect some or all feeding and non-feeding behaviors. For RW, reported correlation coefficients for eating time in both confined and grazing systems range from 0.81 to 0.97 [9,13,16,17]. For ruminating time, most studies report high correlations, typically exceeding 0.90 [9,13,17,18,19,20,21,22], although a few have reported relatively lower values, ranging from 0.68 to 0.76 [16,23,24,25]. In contrast, drinking time generally shows lower accuracy, with most reported correlations around or below 0.50 [9,13,16,19,20,24] and only one study reporting a relatively high value (0.78) [13]. Additionally, despite the strong correlations observed for eating time, overestimation has been reported [9,10,20,26,27,28]. In the case of ND, eating, ruminating, and inactivity times were reported to have strong correlation coefficients of 0.93, 0.92, and 0.84, respectively [14].

Most validation studies for both sensors have been conducted in dairy or beef cattle, although a strong correlation has also been observed for ND in buffaloes [29]. However, no validation studies have been conducted in Hanwoo cattle, a Korean beef breed known for its high marbling. Because Hanwoo are mostly raised in intensive, confined production systems and fed a large amount of concentrate along with low-quality roughage (i.e., rice straw), the performance of these sensors may vary under this type of production system. Furthermore, previous validation studies often used data collected during a limited time frame within a day, rather than covering the entire 24 h period, and no direct comparisons of the two sensors have been conducted. A direct comparison will minimize trial- and case-specific variation, helping to identify the better sensor.

Therefore, this study aimed to evaluate the agreement and reliability of feeding and non-feeding behaviors, particularly eating and ruminating times, recorded by the RW and ND, compared with those obtained through video observation (VO) in Hanwoo cows. The VO is often used as a reference method in behavioral validation studies [9,19,23,26,27].

## 2. Materials and Methods

This study was conducted at the Center for Animal Science Research, Chungnam National University, Republic of Korea. The use of animals and protocols for this experiment were reviewed and pre-approved by the Chungnam National University Animal Research Ethics Committee (202402A-CNU-029).

### 2.1. Animal and Management

Three primiparous, non-pregnant and non-lactating Hanwoo cows (540 ± 24.12 kg body weight [BW]) were used in this study. The cows were housed in the same pen (approximately 50 m^2^), equipped with two feed bunks that identified each cow using radio frequency identification and measured individual feed intake and two automatic water troughs. The pen floor was made of concrete and covered with sawdust as bedding.

Feeding was carried out twice daily (08:00 and 18:00). Tall fescue hay (approximately 6 kg/day per cow) was offered in feed bunks, while concentrate mix (approximately 2 kg/day per cow) was provided on the floor outside the pen but within reach of the cows at the stanchion.

Prior to the onset of the trial, the cows had already been fitted with the ND tags; therefore, no adaptation period was needed. However, the RW was newly fitted to each cow, and thus, sensor attachment and a one-day adjustment period were implemented following the procedure described by Ruuska et al. [9].

### 2.2. Sensor Technologies

#### 2.2.1. RumiWatch Noseband Sensor (RW)

The RW consists of a vegetable oil-filled silicone tube equipped with a pressure sensor and mounted within the casing of a halter positioned over the cow’s nasal bridge. Jaw movements alter the curvature of the tube, resulting in pressure fluctuations within the silicone tube. These pressure variations are detected by the sensor and transmitted to a data logger housed in a protective casing on the right side of the halter, whereas the power supply is located in a separate casing on the left side. The data logger records pressure signals at a constant sampling frequency of 10 Hz and stores the raw data on an SD memory card. When properly formatted, the memory card enables continuous data collection for up to four months [30]. Feeding behavior is subsequently evaluated using two software programs, RumiWatch Manager and RumiWatch Converter, as described by [31]. The pressure sensor detects individual jaw movements as pressure peaks exceeding a 28 mbar threshold, with each peak counted as a chew. Behavioral classification is then based on the frequency and intervals of these peaks, as rumination, eating, drinking, and other activities exhibit distinct patterns. Raw data from the noseband sensor can be transferred to a computer either wirelessly or via the SD memory card using RumiWatch Manager [30].

In the present study, RumiWatch Manager (version 2) was used to manually retrieve data from the noseband through a universal serial bus (USB) connection. Behavioral classification was then performed using RumiWatch Converter (RWC; version 7.3.36; Itin + Hoch GmbH, Liestal, Switzerland), specialized software designed for user-defined post-processing of RumiWatch measurement data. The RWC applies algorithm-based analyses to convert pressure signals into categorized animal activity data. Based on characteristic noseband pressure patterns, ingestive behavior is classified into four categories: rumination, eating, drinking, and other activities [13].

#### 2.2.2. Nedap SmartTag Neck (ND)

The ND is a neck-mounted sensor designed to monitor feeding and activity behaviors in cattle. The device detects eating, ruminating, and inactivity based on characteristic movement patterns of the head and jaw. Rumination is identified from the periodic jaw movements associated with regurgitation and subsequent cud chewing, which occur during both standing and lying positions. Eating behavior is determined from specific head movements associated with feed intake. Inactivity is recorded when the cow exhibits minimal movement and may occur while the animal is either in standing or lying positions (Borchers et al., 2021) [14]. Behavioral data are summarized by the system as 24 h summaries and recorded on a daily basis. Because the ND system provides behavioral data as daily 24 h summaries, hourly-level data were not available, preventing direct comparison between ND and video observation (VO) at the 1 h level. These data are automatically transmitted to a central server from which they are subsequently downloaded.

### 2.3. Video Observation (VO)

Video recordings were conducted for three consecutive days, with continuous 24 h observation of each cow per day. Two closed-circuit television (CCTV) cameras were installed to monitor the cows within the pen. The first camera was positioned approximately 2 m above the cows and about 3 m from the pen, facing the feed bunks and water trough area. The second camera was installed opposite the first at a height of approximately 2 m and a distance of about 6 m from the pen. This configuration ensured a clear and unobstructed view of all cows within the pen. To facilitate nighttime observations, two lamps were installed to provide adequate illumination, allowing continuous monitoring of animal activities throughout the 24 h observation period.

Feeding behavior was evaluated from recorded video footage using an ethogram describing eating, ruminating, drinking, and other activities, compiled from various studies [9,14,32,33,34] (Table 1). All video analyses were conducted by the same observer to ensure consistency. Behavioral durations were determined using the timestamps from the video recordings by calculating the difference between the start and end times of each activity. Because individual cows performed multiple activities throughout the observation period, all behavioral events were recorded according to the behavioral coding scheme for VO established in this study (Table 2). Observation of each cow began at 00:00 h on the day of recording and ended at 00:00 h on the following day, yielding a complete 24 h observation period. A total of three 24 h observations were conducted for each cow. Prior to the start of the experiment, the internal clocks of all sensors and video cameras were synchronized to ensure accurate temporal alignment between sensor data and video observations.

### 2.4. Data Preparation

Based on the coding scheme for VO (Table 2), a data recording sheet was created in Microsoft Excel to generate 1 h and 24 h summaries of behavioral variables, including eating time (ET), ruminating time (RT), drinking time (DT), and inactivity time (IT). In some instances, cows exhibited jaw movements resembling chewing activity that were not associated with feed intake. These movements may be misclassified as eating behavior by the RW, resulting in an overestimation of eating time in previous studies [9,10,17,20,26,27,28]. To account for this, we recorded these as ‘other jaw movements’ (Table 2) and summed the time spent on eating and on other jaw movements as EatOther in the VO data. Agreement between EatOther and ET estimated by each sensor was subsequently evaluated. In addition, adjusted datasets (e.g., ET (adjusted)) were created to improve comparability between RW and VO measurements. Specifically, records were excluded from both RW and VO datasets when VO data indicated zero duration for a given behavior within a 1 h observation period, regardless of the corresponding RW measurement. Comparisons using adjusted data were limited to RW and VO measurements for eating, ruminating, and drinking times. Consequently, behavioral data obtained from RW (min, s) included ET, RT, and DT (1 h and 24 h summaries), whereas those obtained from ND (min, s) included ET, RT, and IT (24 h summary).

### 2.5. Statistical Analysis

An a priori power analysis based on the correlation coefficients reported for rumination time in previous validation studies of the RumiWatch system indicated that, assuming a conservative PCC of 0.80, a sample size of nine paired observations would provide >80% power to detect a significant correlation. Agreement between VO and sensor-derived data was evaluated using the concordance correlation coefficient (CCC), the Pearson correlation coefficient (PCC), and the Spearman correlation coefficient (SCC). The CCC was used to assess both precision and accuracy between measurement methods. The PCC was used to assess the strength of the linear association between measurements, whereas the SCC was used to assess the strength of their monotonic association based on ranked data, particularly when the relationship may not be strictly linear. Values of CCC were interpreted as follows: 0.0–0.3 (negligible), 0.3–0.5 (low), 0.5–0.7 (moderate), 0.7–0.9 (high), and 0.9–1.0 (very high) [35]. The Pearson and Spearman correlation coefficients were interpreted according to the following criteria: <0.3 (poor), 0.3–0.5 (fair), 0.6–0.8 (moderate), 0.8–0.9 (very strong), and 1.0 (perfect), as described by Chan [36]. Negative PCC and SCC values were interpreted using the same magnitude thresholds but indicated inverse relationships between variables [36]. To compare behavioral data obtained using different methods (i.e., VO, RW, and ND), a linear model with the method as fixed effect was used to compute least squares means and perform pairwise comparisons to identify significant differences [37]. Differences between VO and corresponding sensor estimates were calculated to quantify deviations. These deviations were further analyzed to evaluate variation among cows and across observation days using one-way and two-way analyses of variance [37].

## 3. Results

### 3.1. Correlation Between RW and ND with VO

In the 24 h summary (Table 3), correlation coefficients between VO and sensor-estimated behaviors varied according to behavior type and sensor. Between the two sensor systems, RW consistently demonstrated higher correlation coefficients than ND.

For ET, RW showed low positive correlations with VO, ranging from 0.05 (CCC) to 0.50 (SCC), whereas ND showed negative coefficients across all correlation measures, ranging from −0.15 (CCC) to −0.53 (SCC). When EatOther, defined as the combined time spent on both eating and other jaw movements measured by VO, was compared with ET estimated by the sensors, correlation coefficients increased. For RW, coefficients ranged from 0.52 (SCC) to 0.65 (PCC). For ND, correlation coefficients ranged from 0.03 (CCC) to 0.45 (SCC).

In contrast to ET, both sensors showed positive and strong correlation coefficients for RT. Particularly, RW exhibited substantial to very strong agreement with VO, with coefficients ranging from 0.92 (SCC) to 0.95 (CCC and PCC). The ND, on the other hand, showed a low CCC (0.124) but strong correlations with SCC (0.80) and PCC (0.83).

For non-feeding behaviors, correlation coefficients between the biosensors and VO were generally low. For DT, RW showed negative correlations with VO, with coefficients ranging from −0.54 (CCC) to −0.71 (SCC). For IT, ND showed low correlation coefficients with VO, ranging from 0.10 (CCC) to 0.33 (PCC).

In the 1 h summary (Table 4), comparisons between RW and VO indicated higher correlation coefficients for ET and RT than those observed in the 24 h summary, with the exception of DT. For ET, a pattern similar to that observed in the 24 h summary was noted, with the EatOther variable showing enhanced correlations with VO. The RT consistently demonstrated very strong correlations, with coefficients ranging from 0.969 to 0.978, exceeding those observed for ET and DT.

When adjusted data were applied in the 1 h summary, correlation coefficients for ET and EatOther decreased slightly, whereas correlations for RT were slightly enhanced. In contrast, DT continued to show low correlation coefficients with VO, which further decreased after data adjustment.

### 3.2. Mean Comparison of RW, ND, and VO

Mean daily duration (min/d) of feeding and non-feeding behaviors differed among RW, ND, and VO, with RW generally showing agreement with VO whereas ND tended to underestimate behavioral durations (Table 5). For feeding behaviors, ET estimated by both sensors differed significantly from VO (*p* < 0.001). Specifically, RW overestimated ET by 167 min/24 h, whereas ND underestimated ET by 112 min/24 h. The combined parameter representing time spent on both eating and other jaw movements (EatOther) in VO yielded values comparable to ET recorded by RW (*p* > 0.05), whereas ND measurement remained significantly lower by 266 min/24 h (*p* < 0.001). A similar pattern was observed for RT. The RW measurements were comparable to VO (*p* > 0.05), whereas ND significantly underestimated RT by 250 min/24 h (*p* < 0.001). For non-feeding behaviors, DT recorded by RW did not differ from VO (*p* > 0.05). The IT recorded by ND, however, was significantly lower than VO by 147 min/24 h (*p* < 0.001).

**Figure 1:**
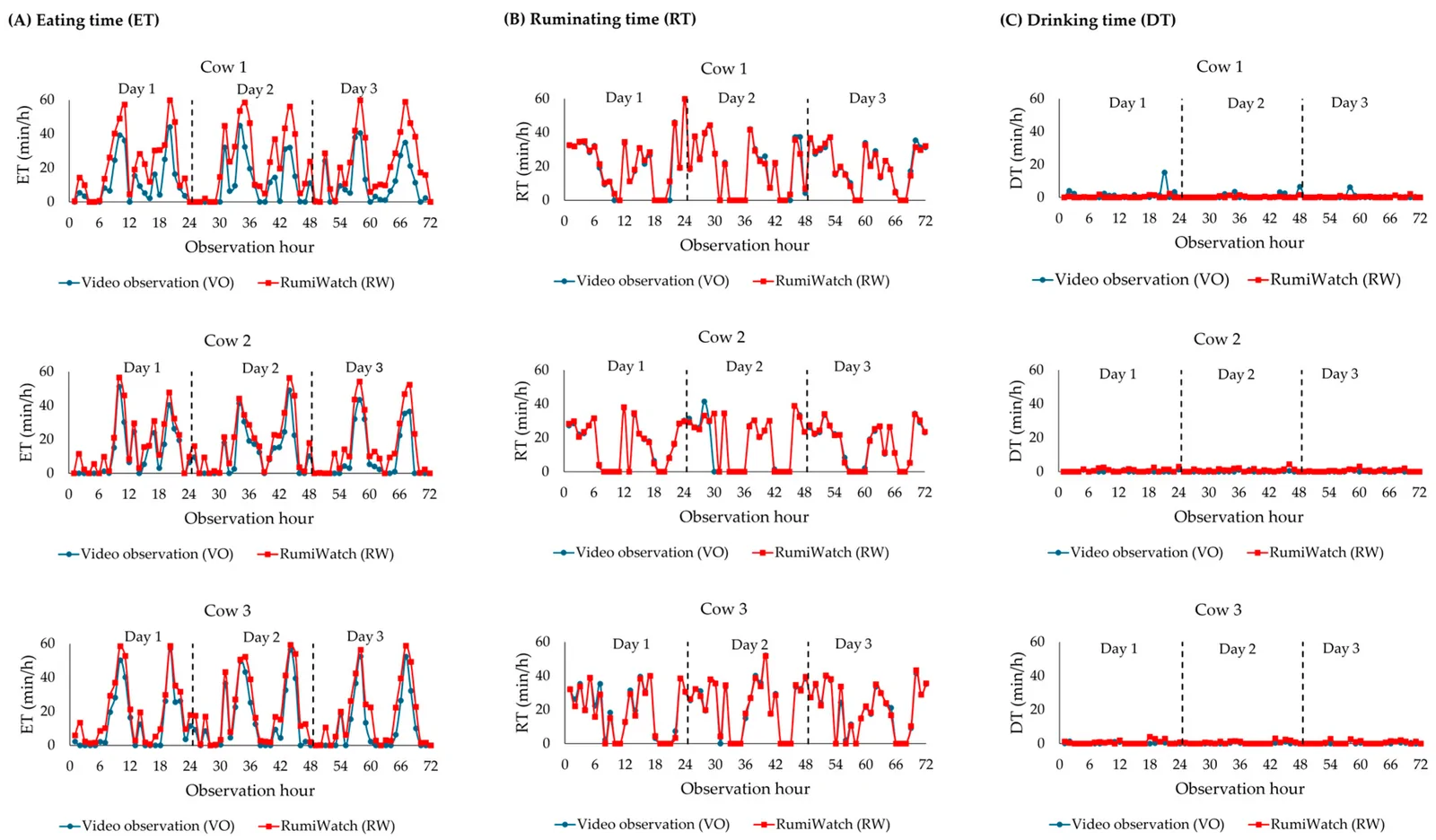
Time series of 1 h summaries of (**A**) eating time (ET), (**B**) ruminating time (RT), and (**C**) drinking time (DT), expressed in min/h, measured by video observation (VO) and the RumiWatch (RW) sensor over three consecutive days (72 h) for each of the three cows. Vertical dashed lines indicate the boundaries between observation days.

Behavioral patterns derived from the 1 h summary were generally consistent with those observed in the 24 h summary, although some discrepancies were noted for non-feeding behaviors (Table 6). For feeding behaviors, ET and ET (adjusted) differed significantly from VO (*p* < 0.001). In contrast, EatOther and EatOther (adjusted) were comparable to VO (*p* > 0.05). Similarly, rumination behavior showed consistent agreement with VO. Both RT and RT (adjusted) were comparable to VO (*p* > 0.05). For non-feeding behaviors, however, the results were not fully consistent with the correlation analysis. Although the correlation analysis indicated weaker agreement, both DT and DT (adjusted) were comparable to VO (*p* > 0.05). To complement these mean comparisons, the time series plots illustrate how the measurements from VO and RW varied over the 72 h observation period for each cow (Figure 1). The RW frequently recorded longer ET than VO across cows, including during some hours when VO recorded no eating, whereas RT profiles showed close correspondence between methods across cows and days. The DT was low during most observation hours for both VO and RW, with occasional differences between methods in the magnitude of hourly measurements, particularly in Cow 1.

### 3.3. Variation in Sensor Measurement Deviations by Cow and Day

Variation in sensor measurements relative to VO in the 24 h summary differed between RW and ND, with ND showing greater influence of the cow factor on feeding-related behaviors (Table 7). For RW measurements, ET, EatOther, RT, and IT were not influenced by either cow or day factors (*p* > 0.05). Only DT was significantly affected by the cow factor (*p* < 0.01). For ND measurements, however, the cow factor significantly affected several feeding-related parameters. Specifically, ET (*p* < 0.01), EatOther (*p* < 0.05), and RT (*p* < 0.01) were significantly influenced by the cow factor, whereas IT was not affected by either cow or day (*p* > 0.05).

In the 1 h summary, deviations of RW measurements from VO were mainly influenced by the cow factor, while the day factor showed no effect across behavioral parameters (Table 8). Specifically, ET, ET (adjusted), EatOther, EatOther (adjusted), DT, and DT (adjusted) were all significantly affected by the cow factor (*p* < 0.0001). In contrast, RT and RT (adjusted) were not influenced by the cow factor (*p* > 0.05). The day factor did not affect deviations of RW measurement from VO for any behavior parameter (*p* > 0.05). However, RT (adjusted) showed a significant interaction between cow and day (*p* < 0.0001).

## 4. Discussion

### 4.1. Agreement of Sensors with VO

Feeding behaviors such as eating and ruminating are essential indicators of cattle productivity, health status, and feed intake, making accurate monitoring of these behaviors important for both research and precision livestock management. With the increasing adoption of wearable biosensors, systems such as RumiWatch noseband and Nedap SmartTag Neck have been widely used to automatically record feeding-related behaviors. However, most previous validation studies evaluating these sensors relied on short observation summaries, typically ranging from minutes to one hour. As a result, sensor performance under longer observation periods, such as a full 24 h behavioral cycle, remains insufficiently evaluated. Furthermore, validation studies have primarily focused on dairy or beef cattle in Western production systems, and no validation has been conducted in Hanwoo cattle.

Agreement between the sensors and VO differed according to sensor type and behavior, with RW generally showing stronger alignment with VO than ND. For eating behavior, RW showed more favorable agreement with VO than ND. In the 24 h summary, RW exhibited positive correlations with VO, whereas ND showed negative correlations across all correlation measures. Previous studies evaluating RW performance reported high correlations for ET, including SCC values ranging from 0.86 to 0.96 [13], a PCC of 0.97 [9], and a CCC of 0.91 [17]. Similarly, studies assessing ND performance reported correlations generally exceeding 0.90 in cattle [14,38,39], with CCC and PCC values of 0.85 and 0.91, respectively, reported in buffalo [29]. These high correlations contrast with the lower correlations observed in the present study. However, correlations for RW increased when data were summarized at 1 h intervals, suggesting that summary duration may influence correlation outcomes. One possible explanation is the misclassification of other jaw movements as eating behavior by RW [22]. During short observation intervals, misclassification events may be dispersed across time frames, whereas in longer summaries, they may accumulate and reduce overall correlations. In contrast, ND data were available only for the 24 h summary in the present study, which prevented evaluation of similar temporal effects. Nevertheless, the negative associations observed for ND indicate substantial disagreement with VO. These differences likely arise from variations in sensor algorithms, device design, and sensor placement. Overall, RW showed greater agreement with VO for ET estimation than ND, and the effect of summary duration suggests that temporal aggregation may influence correlation outcomes when misclassifications occur.

For rumination behavior, both sensors showed stronger agreement with VO than observed for ET. RW consistently exhibited very strong correlations with VO, whereas ND showed lower concordance. Previous validation studies for RW have generally reported correlations ranging from 0.90 to as high as 0.99 for RT when summarized over short time intervals. Slightly lower correlations have also been reported in some studies, including CCC values ranging from 0.76 to 0.91 in calves [23] and correlations ranging from 0.68 to 0.97 after algorithm correction [25]. In grazing systems, validation studies reported slightly lower correlations, such as a SCC of 0.81 [16] and a CCC of 0.83 with a PCC of 0.94 [24]. For ND, previous studies reported higher correlations than those observed in the present study, with CCC values ranging from 0.89 to 0.92 when summarized over short intervals [14,29,39]. Unlike ET, correlations for RT in RW remained consistently high (>0.90) across both 1 h and 24 h summaries, indicating that RT estimation was not affected by summary duration. This stability may be attributable to the behavioral characteristics of rumination, which consists of continuous and repetitive jaw movements that are more easily detected than eating behavior [9]. Additionally, the placement of RW around the nose and jaw allows direct detection of jaw movements, whereas ND relies primarily on head and neck movement patterns. These methodological differences likely contributed to the stronger agreement observed for RW. Collectively, RT estimation by RW closely aligned with VO and remained robust across summary durations.

For non-feeding behaviors, both sensors exhibited substantially weaker agreement with VO compared with feeding behaviors. The DT recorded by RW showed predominantly negative correlations with VO, whereas IT recorded by ND showed low correlations that did not exceed 0.33. Previous studies evaluating RW for DT have similarly reported low correlations [9,13,16,19,20,24]. Drinking behavior is inherently difficult to detect accurately because it occurs sporadically and typically lasts for short durations. Cattle usually drink only a few times per day, and these brief events reduce the likelihood of correct classification [9,13,16,20,24]. Furthermore, drinking behavior may share movement characteristics with other oral or feeding-related activities, increasing the likelihood of misclassification. Pereira et al. [24], for example, reported cases in which drinking events were detected by sensors without visual confirmation, indicating that similar behaviors may be incorrectly classified as drinking. For inactivity behavior, ND also showed weak agreement with VO despite similar behavioral definitions during VO; cows classified as inactive occasionally performed brief actions such as licking themselves or nearby cows or performing subtle oral movements while remaining stationary. Although these activities were categorized as inactivity during visual observation, ND may have interpreted these movements differently because classification relies on head and jaw movement patterns. Consequently, these brief activities may have been excluded from inactivity or assigned to other behaviors by the sensor. Taken together, these results suggest that non-feeding behaviors are inherently more difficult to classify using sensor-based systems and therefore show weaker agreement with VO compared with feeding behaviors.

### 4.2. Measurement Differences Between Sensors and VO

In addition to differences in agreement, both sensors exhibited systematic measurement bias relative to VO, particularly for ET. The RW tended to overestimate ET, whereas ND underestimated ET. In the 24 h summary, RW recorded significantly higher ET values than both VO and ND, while ND recorded significantly lower ET values than both RW and VO. When the EatOther parameter from VO was used for comparison, RW estimates became comparable to VO, whereas ND remained significantly lower. A similar pattern was observed in the 1 h summary. Furthermore, analysis using adjusted data showed that ET (adjusted) recorded by RW remained significantly greater than VO, indicating that RW frequently recorded longer eating durations within individual time frames. Variation analysis further indicated that ET deviations were influenced by the cow factor for both sensors, suggesting that the magnitude of overestimation or underestimation varied among animals.

Overestimation of ET by RW has been reported in several studies [9,10,20,26,27,28]. Multiple factors may contribute to this phenomenon. First, video observation may fail to capture some chewing events, particularly when they occur rapidly [26]. Second, non-ingestive oral behaviors such as licking may be incorrectly classified as eating by the sensor [28]. Third, loose housing environments may increase mouth-related behaviors that are incorrectly classified as eating [9]. Although the cows in the current study were housed in a confined pen, they still had sufficient space and opportunities to perform other oral activities. Fourth, differentiating eating chews from other jaw movements is inherently more complex than identifying rumination patterns [9,20]. Fifth, the sensitive noseband pressure sensor may record jaw-related movements associated with walking or other head movements [22]. Additional factors include time sequences not clearly assigned to rumination or other activities [40] and abrupt transitions between behaviors, such as when cows switch from rumination to eating while approaching the feeding area, which was also observed in the present study. In contrast, previous studies evaluating ND reported little or no systematic bias in ET estimation [14,29,38], which differs from the underestimation observed in the present study. Nevertheless, the relatively low mean ET values recorded by ND were consistent with the negative correlations observed in the agreement analysis. These findings indicate that although both sensors can detect eating activity, systematic deviations from VO remain, requiring careful interpretation of ET values.

For rumination behavior, RW provided more accurate estimates than ND. The RW estimates of RT were comparable to VO in both 24 h and 1 h summaries, and deviations between RW and VO were not influenced by cow or day factors. In contrast, ND recorded significantly lower RT than both VO and RW in the 24 h summary, and deviations varied significantly among cows. Previous studies evaluating RW performance have reported minor mean deviations from reference methods and no evidence of systematic underestimation or overestimation of RT [13,21,22,24]. Although overestimation of rumination has occasionally been reported, this has generally been associated with chew counts rather than total ruminating time [18,26]. Previous ND validation studies also reported no significant bias for RT estimation based on the Bland–Altman analysis [14,29], which contrasts with the underestimation observed in the present study. Differences in summary duration and sensor limitations may partially explain these discrepancies. Overall, the mean comparison results were consistent with the correlation analysis, confirming that RW provided more reliable RT estimates than ND.

For non-feeding behaviors, RW produced mean DT values comparable to VO, whereas ND underestimated IT. Mean DT did not differ from VO in either the 24 h or 1 h summaries, whereas mean IT recorded by ND was significantly lower than VO. Previous studies evaluating RW for DT estimation reported small mean deviations based on the Bland–Altman analysis [13,24] and comparable mean DT relative to direct observation [16], which is consistent with the present findings. However, comparable mean values do not necessarily indicate accurate measurement. In the present study, RW showed low correlations with VO and significant cow-related variation in DT deviations, indicating inconsistent detection of drinking events. As discussed earlier, RW may occasionally detect drinking behavior even when visual observation does not confirm actual drinking activity, which may contribute to this discrepancy. In contrast, previous studies evaluating ND reported mean IT values comparable to reference standards with minimal bias [14]. This contrasts with the present results, where ND significantly underestimated IT. Notably, this underestimation was consistent across cow and days and was supported by weak correlations with VO. These findings indicate that mean equivalence alone is insufficient to infer measurement accuracy and highlight the challenges associated with detecting non-feeding behaviors using sensor-based systems.

Overall, the results of the present study demonstrate that the performance of sensor-based behavioral monitoring systems varies substantially according to both sensor type and behavior category. The RW showed stronger agreement with VO than ND for most feeding behaviors, particularly rumination, whereas ND exhibited lower correlations and greater variability across animals. These findings indicate that sensor design and placement can strongly influence the accuracy of behavioral classification in cattle.

Despite the insights provided by this study, several limitations should be acknowledged. First, the number of animals included in this study was limited. Although repeated measurements within individuals over the entire 24 h period across three consecutive days increased the number of observations and improved the precision of within-animal comparisons, they did not increase the number of independent experimental units. Therefore, the limited number of animals restricts the generalizability of the findings to broader populations or different production conditions. Nevertheless, the repeated observations allowed the consistency of sensor outputs relative to visual observations to be evaluated across individuals and days. Further validation using a large number of animals and more diverse production conditions is warranted to confirm the applicability and reliability of these findings. In addition, ND data were available only in a 24 h summary format, which prevented evaluation of how the summary duration might influence correlations and measurement deviations for this sensor.

Future studies should evaluate sensor performance across multiple summary durations and production environments to better understand how temporal aggregation influences behavioral classification accuracy. Additionally, algorithm refinement may improve detection of short-duration or sporadic behaviors such as drinking and inactivity. Future research could also explore the integration of complementary sensing technologies or multimodal data streams to improve behavioral classification accuracy. For example, combining multiple behavioral indicators or sensor systems may enhance the detection of complex activities and reduce misclassification.

## 5. Conclusions

In conclusion, RW demonstrated stronger agreement with visual observation than ND for most feeding behaviors, particularly rumination behavior. While RW tended to overestimate eating time and ND tended to underestimate several behavioral measures, ruminating time recorded by RW remained closely aligned with visual observations across summary durations. These findings indicate that RW provides more reliable estimates of rumination behavior, whereas both sensors require further refinement of their algorithms to improve the accuracy of eating and non-feeding (drinking or inactivity) behavior detection.

## Figures and Tables

**Table 1:** Behavioral ethogram used for identifying eating, drinking, ruminating, and other activities.

Behavior	Definition
Eating	Head positioned over or inside the feed bunk Actively chewing while standing near the feed bunk Taking feed into the mouth, chewing, and swallowing it, regardless of whether the feed was obtained from the floor, feed bunk, or other areas of the pen Cow standing at the feed bunk while chewing and ingesting feed The eating event was classified as finished when chewing ceased for 5 s or more
Drinking	Head positioned over or inside the water trough Cow placing its muzzle into the water bowl and swallowing water
Ruminating	Time the cow regurgitated a bolus, chewed it, and re-swallowed it Commenced with the regurgitation of a bolus followed by rhythmic chewing Cow lying or standing while continuously chewing and showing a vomit-like movement between chewing bouts Cow masticating or swallowing ingesta while sitting or standing
Other activities	Active Walking or engaging in other movements Other chewing activity Chewing activity not classified as eating and ruminating Inactivity Not actively moving, occurring either in standing or lying position

**Table 2:** Behavioral coding scheme for video observation (VO).

Behavior	Code	Description
Walking	Eat1	Eating while walking/moving
	Rum1	Ruminating while walking/moving
	OJ1	Other jaw movement (e.g., licking) while walking/moving
	NJM1	No jaw movement while walking/moving
Standing	NJM2	Cow is inside the feed bunk with no jaw movement
	Eat2	Cow is inside the feed bunk while eating
	Eat3	Cow is eating while standing still outside the feed bunk
	Rum2	Cow is standing still while ruminating
	Rum3	Cow is standing still while ruminating inside feed bunk
	OJ2	Cow is inside the feed bunk showing other jaw movements (e.g., licking)
	OJ3	Cow is standing still with other jaw movement (e.g., licking)
	Drink	Cow is drinking
	NJM3	Cow is standing still with no jaw movement
Lying	Rum4	Ruminating while lying
	OJ4	Other jaw movements while lying (e.g., licking)
	NJM4	No jaw movement while lying

**Table 3:** Correlation coefficient values between video-observed (VO) and sensor-estimated feeding and non-feeding behaviors in 24 h summary (min/24 h).

Parameter ^1^	Comparison ^2^	*n*	Correlation Coefficient ^3^		
			CCC	PCC	SCC
ET	VO-RW	9	0.047	0.360	0.502
	VO-ND	9	−0.149	−0.526	−0.533
EatOther	VO-RW	9	0.619	0.648	0.519
	VO-ND	9	0.030	0.333	0.450
RT	VO-RW	9	0.949	0.949	0.917
	VO-ND	9	0.124	0.830	0.798
DT	VO-RW	9	−0.538	−0.700	−0.713
IT	VO-ND	9	0.103	0.328	0.150

^1^ ET, eating time; EatOther, time spent on eating and other jaw movements; RT, ruminating time; DT, drinking time; IT, inactivity time. ^2^ VO, video observation; RW, Rumiwatch noseband sensor; ND, Nedap smarttag neck sensor. ^3^ CCC, concordance correlation coefficient; PCC, Pearson correlation coefficient; SCC, Spearman correlation coefficient.

**Table 4:** Correlation coefficient values between video-observed (VO) and RW-estimated behaviors using 1 h summary (min/hour).

Parameter ^1^	*n*	Correlation Coefficient ^2^		
		CCC	PCC	SCC
ET	216	0.823	0.929	0.921
ET (adjusted) ^3^	136	0.744	0.908	0.898
EatOther	216	0.972	0.975	0.960
EatOther (adjusted)	194	0.968	0.972	0.958
RT	216	0.978	0.978	0.969
RT (adjusted)	161	0.983	0.984	0.975
DT	216	−0.139	0.021	0.299
DT (adjusted)	83	−0.139	−0.175	−0.156

^1^ ET, eating time; EatOther, time spent on eating and other jaw movements; RT, ruminating time; DT, drinking time. ^2^ CCC, concordance correlation coefficient; PCC, Pearson correlation coefficient; SCC, Spearman correlation coefficient. ^3^ Some records were removed from the datasets when video observations showed zero time during each 1 h frame. Of the original 216 records for each behavior, 80 ET (37.04%), 22 EatOther (10.19%), 55 RT (25.46%), and 133 DT (61.57%) records were removed.

**Table 5:** Mean comparison of video-observed (VO) and sensor-estimated feeding and non-feeding behaviors for 24 h summary (min/24 h).

Parameter ^1^	Method ^2^			SEM	*p*-Value
	VO	RW	ND		
ET	287 ^b^	454 ^a^	175 ^c^	20.4	<0.001
EatOther	441 ^a^	454 ^a^	175 ^b^	21.3	<0.001
RT	461 ^a^	460 ^a^	211 ^b^	28.8	<0.001
DT	12.1	14.5	-	2.81	>0.050
IT	1192	-	1045	30.0	<0.001

^1^ ET, eating time; EatOther, time spent on eating and other jaw movements; RT, ruminating time; DT, drinking time; IT, inactivity time. ^2^ VO, video observation; RW, Rumiwatch noseband sensor; ND, Nedap smarttag neck sensor. ^a–c^ Means within a row with different letters are significantly different (*p* < 0.05). SEM, standard error of the mean.

**Table 6:** Mean comparison of video-observed (VO) and RW-estimated behaviors for 1 h summary (min/h).

Parameter ^1^	Method ^2^		SEM	*p*-Value
	VO	RW		
ET	11.8	19.5	1.14	<0.001
ET (adjusted) ^3^	18.8	29.1	1.35	<0.001
EatOther	18.2	19.5	1.22	>0.050
EatOther (adjusted)	20.3	21.7	1.27	>0.050
RT	19.1	19.1	0.98	>0.050
RT (adjusted)	25.6	25.3	0.86	>0.050
DT	0.447	0.599	0.0764	>0.050
DT (adjusted)	1.164	0.938	0.1710	>0.050

^1^ ET, eating time; EatOther, time spent on eating and other jaw movements; RT, ruminating time; DT, drinking time. ^2^ VO, video observation; RW, Rumiwatch noseband sensor. ^3^ Some records were removed from the datasets when visual observations showed zero time during each 1 h frame. Of the original 216 records for each behavior, 80 ET (37.04%), 22 EatOther (10.19%), 55 RT (25.46%), and 133 DT (61.57%) records were removed. SEM, standard error of the mean.

**Table 7:** Effects of cow and day on deviations between sensor-estimated and video-observed feeding and non-feeding behaviors using 24 h summaries.

Parameter ^1^	Sensor ^2^	Cow	Day
ET	RW	n.s.	n.s.
	ND	**	n.s
EatOther	RW	n.s.	n.s.
	ND	*	n.s.
RT	RW	n.s.	n.s.
	ND	**	n.s.
DT	RW	**	n.s.
IT	ND	n.s.	n.s.

^1^ ET, eating time; EatOther, time spent on eating and other jaw movements; RT, ruminating time; DT, drinking time; IT, inactivity time. ^2^ RW, rumiwatch noseband sensor; ND, nedap smarttag neck sensor. n.s., non-significant (*p* > 0.05); *, *p* < 0.05; **, *p* < 0.01.

**Table 8:** Two-way ANOVA of deviations between RumiWatch noseband sensor (RW)-estimated and video-observed (VO) feeding and non-feeding behaviors using 1 h summaries, with cow, day, and their interaction as factors.

Parameter ^1^	Cow	Day	Cow * Day
ET	***	n.s.	n.s.
ET (adjusted) ^2^	***	n.s.	n.s.
EatOther	***	n.s.	n.s.
EatOther (adjusted)	***	n.s.	n.s.
RT	n.s.	n.s.	n.s.
RT (adjusted)	n.s.	n.s.	***
DT	***	n.s.	n.s.
DT (adjusted)	***	n.s.	n.s.

^1^ ET, eating time; EatOther, time spent on eating and other jaw movements; RT, ruminating time, DT, drinking time. ^2^ Some records were removed from the datasets when visual observations showed zero time during each 1 h frame. Of the original 216 records for each behavior, 80 ET (37.04%), 22 EatOther (10.19%), 55 RT (25.46%), and 133 DT (61.57%) records were removed. n.s., non-significant (*p* > 0.05); *, *p* < 0.05; ***, *p* < 0.001.

## Data Availability

Data are available from the corresponding author upon reasonable request.

## References

[1] Campling R.C., Morgan C.A. (1981). Eating behaviour of housed dairy cows—A review. Dairy Sci. Abstr..

[2] Codl R., Ducháček J., Vacek M., Pytlík J., Stádník L., Vrhel M. (2023). The influence of eating and rumination time on solids content in milk and milk yield performance of cows. Czech J. Anim. Sci..

[3] Beauchemin K.A. (2018). Invited review: Current perspectives on eating and rumination activity in dairy cows. J. Dairy Sci..

[4] Metz J.H.M. (1975). Time Patterns of Feeding and Rumination in Domestic Cattle. Ph.D. Dissertation.

[5] Bernhard J.K., Stucki D., Achermann R.L., Rediger R., Fadul M., Steiner A. (2021). The lying and rumination behavior of healthy dairy cows housed in tie stalls is associated with bedding depth. Schweiz. Arch. Tierheilkd..

[6] Paudyal S. (2021). Using rumination time to manage health and reproduction in dairy cattle: A review. Vet. Q..

[7] Gill J., Campling R.C., Westgarth D.R. (1966). A study of chewing during eating in the cow. Br. J. Nutr..

[8] Penning P.D. (1983). A technique to record automatically some aspects of grazing and ruminating behavior in sheep. Grass Forage Sci..

[9] Ruuska S., Kajava S., Mughal M., Zehner N., Mononen J. (2016). Validation of a pressure sensor-based system for measuring eating, rumination and drinking behaviour of dairy cattle. Appl. Anim. Behav. Sci..

[10] Pereira G.M., Heins B.J., O’Brien B., McDonagh A., Lidauer L., Kickinger F. (2020). Validation of an ear tag–based accelerometer system for detecting grazing behavior of dairy cows. J. Dairy Sci..

[11] Berckmans D. (2017). General introduction to precision livestock farming. Anim. Front..

[12] Halachmi I. (2015). Precision Livestock Farming Applications: Making Sense of Sensors to Support farm Management.

[13] Zehner N., Umstätter C., Niederhauser J.J., Schick M. (2017). System specification and validation of a noseband pressure sensor for measurement of ruminating and eating behavior in stable-fed cows. Comput. Electron. Agric..

[14] Borchers M.R., Gavigan S., Harbers A., Bewley J. (2021). An evaluation of a novel device for measuring eating, rumination, and inactive behaviors in lactating Holstein dairy cattle. Animal.

[15] Hut P.R., Mulder A., Broek J.V.D., Hulsen J.H.J.L., Hooijer G.A., Stassen E.N., Van Eerdenburg F.J.C.M., Nielen M. (2019). Sensor based eating time variables of dairy cows in the transition period related to the time to first service. Prev. Vet. Med..

[16] Poulopoulou I., Lambertz C., Gauly M. (2019). Are automated sensors a reliable tool to estimate behavioural activities in grazing beef cattle?. Appl. Anim. Behav. Sci..

[17] Weinert-Nelson J.R., Jacobs A.A., Werner J., Williams C.A., Davis B.E. (2024). Impacts of heat stress on the accuracy of a noseband sensor for detection of eating and rumination behavior in confined cattle. JDS Commun..

[18] Kröger I., Humer E., Neubauer V., Kraft N., Ertl P., Zebeli Q. (2016). Validation of a noseband sensor system for monitoring ruminating activity in cows under different feeding regimens. Livest. Sci..

[19] Li Z., Cheng L., Cullen B. Assessment of grazing, rumination, and other activities in dairy cow by using RumiWatch noseband sensor in automatic milking system. Proceedings of the Australasian Dairy Science Symposium 2022.

[20] Steinmetz M., Soosten D., Hummel J., Meyer U., Dänicke S. (2020). Validation of the RumiWatch converter V0.7.4.5 classification accuracy for the automatic monitoring of behavioural characteristics in dairy cows. Arch. Anim. Nutr..

[21] Werner J., Lesoa L., Umstatter C., Niederhauser J., Kennedya E., Geoghegan A., Shalloo L., Schick M., O’Briena B. (2018). Evaluation of the RumiWatch system for measuring grazing behaviour of cows. J. Neurosci. Methods.

[22] Werner J., Umstatter C., Leso L., Kennedy E., Geoghegan A., Shalloo L., Schick M., O’Brien B. (2019). Evaluation and application potential of an accelerometer-based collar device for measuring grazing behavior of dairy cows. Animal.

[23] Eslamizad M., Tümmler L.-M., Derno M., Hoch M., Kuhla B. (2018). TECHNICAL NOTE: Development of a pressure sensor-based system for measuring rumination time in pre-weaned dairy calves. J. Anim. Sci..

[24] Pereira G.M., Sharpe K.T., Heins B.J. (2021). Evaluation of the RumiWatch system as a benchmark to monitor feeding and locomotion behaviors of grazing dairy cows. J. Dairy Sci..

[25] Pichlbauer B., Chapa Gonzalez J.M., Bobal M., Guse C., Iwersen M., Drillich M. (2024). Evaluation of different sensor systems for classifying the behavior of dairy cows on pasture. Sensors.

[26] Li Z., Cheng L., Cullen B. (2021). Validation and use of the RumiWatch noseband sensor for monitoring grazing behaviours of lactating dairy cows. Dairy.

[27] Norbu N., Alvarez-Hess P.S., Leury B.J., Wright M.M., Douglas M.L., Moate P.J., Williams S.R.O., Marett L.C., Garner J.B., Wales W.J. (2021). Assessment of RumiWatch noseband sensors for the quantification of ingestive behaviors of dairy cows at grazing or fed in stalls. Anim. Feed Sci. Technol..

[28] Raynor E.J., Derner J.D., Soder K.J., Augustine D.J. (2021). Noseband sensor validation and behavioural indicators for assessing beef cattle grazing on extensive pastures. Appl. Anim. Behav. Sci..

[29] Quddus R.A., Ahmad N., Khalique A., Bhatti J.A. (2022). Validation of NEDAP monitoring technology for measurements of feeding, rumination, lying, and standing behaviors, and comparison with visual observation and video recording in buffaloes. Animals.

[30] Zehner N., Niederhauser J.J., Nydegger F., Grothmann A., Keller M., Hoch M., Haeussermann A., Schick M. Validation of a new health monitoring system (RumiWatch) for combined automatic measurement of rumination, feed intake, water intake and locomotion in dairy cows. Proceedings of the International Conference of Agricultural Engineering CIGR.

[31] Zehner N. (2015). RumiWatch Manager, version 1.0.0.1.

[32] Ambriz-Vilchis V., Jessop N.S., Fawcett R.H., Shaw D.J., Macrae A.I. (2015). Comparison of rumination activity measured using rumination collars against direct visual observations and analysis of video recordings of dairy cows in commercial farm environments. J. Dairy Sci..

[33] Ayadi S., Ben Said A., Jabbar R., Aloulou C., Chabbouh A., Achballah A.B., Jemili I., Mosbah M. (2020). Dairy Cow Rumination Detection: A Deep Learning Approach. Distributed Computing for Emerging Smart Networks.

[34] Borchers M.R., Chang Y.M., Tsai I.C., Wadsworth B.A., Bewley J.M. (2016). A validation of technologies monitoring dairy cow feeding, ruminating, and lying behaviors. J. Dairy Sci..

[35] Hinkle D.E., Wiersma W., Jurs S.G. (2003). Applied Statistics for the Behavioral Sciences.

[36] Chan Y.H. (2003). Biostatistics 104: Correlational analysis. Singap. Med. J..

[37] Seo S., Jeon S., Ha J.K. (2018). Editorial—Guidelines for experimental design and statistical analyses in animal studies submitted for publication in the Asian-Australasian Journal of Animal Sciences. Asian-Australas. J. Anim. Sci..

[38] Dela Rue B., Lee J.M., Eastwood C.R., Macdonald K.A., Gregorini P. (2020). Short communication: Evaluation of an eating time sensor for use in pasture-based dairy systems. J. Dairy Sci..

[39] Kooij E.V., Brug M.V., Roelofs J.B., Kamphuis C., Steeneveld W. (2016). Validation of Nedap SmartTag leg and neck to assess behavioural activity level in dairy cattle. Precision Dairy Farming 2016.

[40] Rombach M., Münger A., Niederhauser J., Südekum K.H., Schori F. (2018). Evaluation and validation of an automatic jaw movement recorder (RumiWatch) for ingestive and rumination behaviors of dairy cows during grazing and supplementation. J. Dairy Sci..

